# Proteostasis networks in aging: novel insights from text-mining approaches

**DOI:** 10.1007/s10522-023-10027-0

**Published:** 2023-04-01

**Authors:** Diogo Neves, Sara Duarte-Pereira, Sérgio Matos, Raquel M. Silva

**Affiliations:** 1 Department of Medical Sciences & iBiMED, University of Aveiro, Aveiro, Portugal; 2 IEETA, University of Aveiro, Aveiro, Portugal; 3 DETI, University of Aveiro, Aveiro, Portugal; 4 Universidade Católica Portuguesa, Faculdade de Medicina Dentária, Centro de Investigação Interdisciplinar em Saúde, Estrada da Circunvalação, 3504-505 Viseu, Portugal

**Keywords:** Protein aggregation, Protein–protein interactions, Inflammasome, NAD metabolism, EGAS

## Abstract

Aging is a topic of paramount importance in an increasingly elderly society and has been the focus of extensive research. Protein homeostasis (proteostasis) decline is a hallmark in aging and several age-related diseases, but which specific proteins and mechanisms are involved in proteostasis (de)regulation during the aging process remain largely unknown. Here, we used different text-mining tools complemented with protein–protein interaction data to address this complex topic. Analysis of the integrated protein interaction networks identified novel proteins and pathways associated to proteostasis mechanisms and aging or age-related disorders, indicating that this approach is useful to identify previously unknown links and for retrieving information of potential novel biomarkers or therapeutic targets.

**Supplementary Information:**

The online version contains supplementary material available at 10.1007/s10522-023-10027-0.

## Introduction

Aging is the progressive physiological and functional decline of organisms that affects the tissues and organs of the whole body. It is best characterized as a multifactorial process that comprises the interaction of cellular and molecular mechanisms, however, individuals do not age at the same rate (Broz and Dixit 2016). Since it is estimated that by 2050, more than 20% of the world´s population will be over 65 years of age (Beard et al. 2016), finding healthy aging biomarkers and therapeutic targets is of outmost importance to predict the biological age of individuals and significantly impact the socio-economic burden caused by an increasingly elderly society.

Maintaining protein homeostasis (proteostasis) is crucial for protein structure, stability, and functional properties. The proteostasis network includes chaperones, co-chaperones, the ubiquitin-proteasome system (UPS) and the autophagic machinery as protein quality control mechanisms (Díaz-Villanueva et al. 2015). In addition to these processes, organelle-specific systems exist and encompass the heat-shock response (HSR), as well as the endoplasmic reticulum (ER) and mitochondria unfolded protein response (UPR^ER^ and UPR^mito^). These processes act in a coordinated fashion to ensure correct protein folding (Díaz-Villanueva et al. 2015; Read and Schröder 2021). When these cellular and molecular protein quality control machineries are overwhelmed, the formation of unfolded proteins increases the tendency of proteins to aggregate.

Earlier in the past decade, *Lopez-Otín *et al*.* included defective proteostasis, or proteotoxicity, in a panel of hallmarks of aging and age-related disorders (López-otín et al. 2013). Despite numerous publications that revealed that unbalanced proteostasis might be a consequence or a cause of aging (Thibaudeau et al. 2018; Luna et al. 2018; Kelmer Sacramento et al. 2020), there is still a lack of proteins that combine these two phenomena. As a better understanding of the molecular pathways involved in proteostasis (de)regulation during aging might lead to the identification of biomarkers and targets for therapeutic intervention, the objective of this work was to provide an integrative view of the regulatory networks involved in proteostasis and the aging process, using two complementary text-mining approaches.

## Material and methods

### Automated text-mining dataset

Automated text mining analysis was performed using the Agilent Literature Search plugin for Cytoscape (Cline et al. 2007). This plugin retrieves documents for a user query and extracts protein–protein associations from these texts using a set of lexicons that define protein names and association terms. A set of queries was built up to include “Proteostasis”, “protein aggregation” and “proteotoxicity” as the terms selected and “aging”, “ageing” and “senescence” as the context query. The species *Homo sapiens*, *Mus musculus*, *Caenorhabditis elegans* and *Drosophila melanogaster* were considered, resulting in the retrieval and analysis of 769 full-text biomedical research papers and the creation of a list of proteins for each of the species. Under extraction controls the option “relaxed” was chosen, which means that a more permissive set of association terms to identify potential protein interactions was considered.

### Curated text-mining dataset

A curated dataset of protein interactions was generated using EGAS (Campos et al. 2014; Matos et al. 2016). Pre-processed abstracts containing automatically identified concepts (proteins, drugs, species, bioprocesses) and relations (protein–protein and protein-chemical interactions) were revised by the curators using the interactive tool. The 1920 results obtained from the PubMed search ((protein aggregation OR proteostasis OR proteotoxicity) AND (ageing OR aging OR senescence)) were prioritized using a linear Support Vector Machine (SVM) classifier. The classifier was initially trained with data from the BioCreative III protein–protein interaction (PPI) article classification task (Krallinger et al. 2011), consisting of 6280 PubMed abstracts classified as containing PPI information (n = 1822) or not (n = 4458). This classifier was then refined through two steps of user feedback in which the 300 highest ranked abstracts, according to classification probability, were manually verified and annotated as containing relevant information (positive) or otherwise (negative). After retraining the classifier using these manually verified documents the full list of 1920 PubMed results was re-ranked. Curators then annotated the first documents of this prioritized list, resulting in a curated corpus of 108 abstracts. Specifically, this annotation process was performed using EGAS, a web-based text-mining and assisted curation tool. EGAS uses dictionary matching and machine learning to automatically identify and annotate concepts and concept relations mentioned in text document. These automatic annotations were then inspected, corrected or removed manually by curators, who also added new annotations. After exporting the curated annotations to a tabular text format, a manual revision step was required to assign unique UniProt identifiers (47 IDs) to protein annotations that had not been normalized during the interactive curation step. The final PPI pairs were imported to Cytoscape for network visualization.

### Network analysis

Cytoscape version 3.4.0 (Shannon et al. 2003) was used to visualize and analyze the protein networks resulting from the two text-mining approaches. A third network was generated using the Cytoscape “import—network—from public databases” tool. The list of UniProt accession IDs was used as input to retrieve the protein–protein interaction (PPI) network from databases of the IMEX consortium (Orchard et al. 2012) (IntAct, MINT, I2D, InnateDB, MPIDb). The human proteins were selected and the PPIs from the two text-mining approaches that were not present in databases were identified. For each network, self-loops and duplicated edges were removed and a column was added to the edge table, indicating the number of underlying edges to each interaction. The EGAS and Agilent filtered networks (2NET) were merged, and these were merged with the network built with information retrieved from public databases (3NET). For key proteins (nodes) the first neighbors were selected, and subnetworks were created containing only edges connected to the central node, to evaluate which interactions were identified by each approach. In the merged network of the three approaches, a clustering analysis was performed using MCODE App from Cytoscape (Bader and Hogue 2003). The protein sub-network consisting of the novel protein interactions (excluding databases) was visualized with STRING (http://string-db.org/), using evidence data from text-mining and experiments with a medium confidence score (0.4) and no interactors in the first or second shell.

### Enrichment and pathway analyses

The list of UniProt IDs of each dataset was analyzed in the Reactome Pathway Database (Croft et al. 2011). All non-human identifiers were converted to their human counterparts. The statistical overrepresentation test was performed in PANTHER (www.pantherdb.org), using PANTHER GO-Slim Biological Process dataset (Mi et al. 2017). Gene enrichment analyses were performed with the Functional annotation clustering tool in DAVID (https://david.ncifcrf.gov/), using the default parameters (Sherman et al. 2022; Huang et al. 2009) These analyses provided statistical measures for the association of our datasets to gene ontology terms, giving an insight of the functional biological meaning of the results.

## Results

For the automated text-mining dataset, the Agilent Literature Search plugin for Cytoscape was used to generate a list of proteins for all the species considered. The resulting protein networks consist of 155 nodes (proteins) and 284 edges (interactions) for *D. melanogaster*, 74 nodes and 105 edges for *C. elegans*, 325 nodes and 629 edges for *H. sapiens* and 265 nodes and 466 edges for *M. musculus*. All proteins from each species were analyzed in the Reactome Pathway Database and similar results were obtained for all species. The integrated analysis of the statistically significant processes shows an overrepresentation of pathways related to the immune system, cellular responses to stress and programmed cell death (Supplementary Fig. 1).

The curated text-mining dataset was obtained using EGAS. A total of 110 protein–protein interactions (PPIs), nine protein-chemical interactions (PCIs) and 62 protein-pathway relations were identified, involving 91 unique proteins that were mapped to their UniProt accession IDs. Fifty-six protein names were not mapped and appear as annotated in the text. For network analysis, only PPIs were considered.

The results of the statistical overrepresentation test, performed on the 91 UniProt IDs from the EGAS dataset, showed that the main processes represented were apoptotic process, response to stress, intracellular signal transduction and regulation of biological process. The analysis in the Reactome Pathway Database revealed that the top four pathways represented were related to cellular responses to stress, particularly to heat stress and involving heat shock transcription factor 1 (HSF1). A total of 34 pathways had a significant p-value (< 0.001) and were involved mainly in signal transduction, immune system, programmed cell death and disease. Overall, the results from both automated and curated datasets were similar.

To compare the output obtained from the literature searches and text mining tools with the information described in specific databases of protein–protein interactions, a network using the automatic search of public databases in Cytoscape was created. As an input, the smaller dataset of protein IDs, consisting of 91 UniProt accession numbers obtained from EGAS was used and only the databases from IMEX Consortium, which are curated and standardized, were selected. A network with a total of 5414 interactions involving 3578 proteins was obtained.

**Fig. 1:**
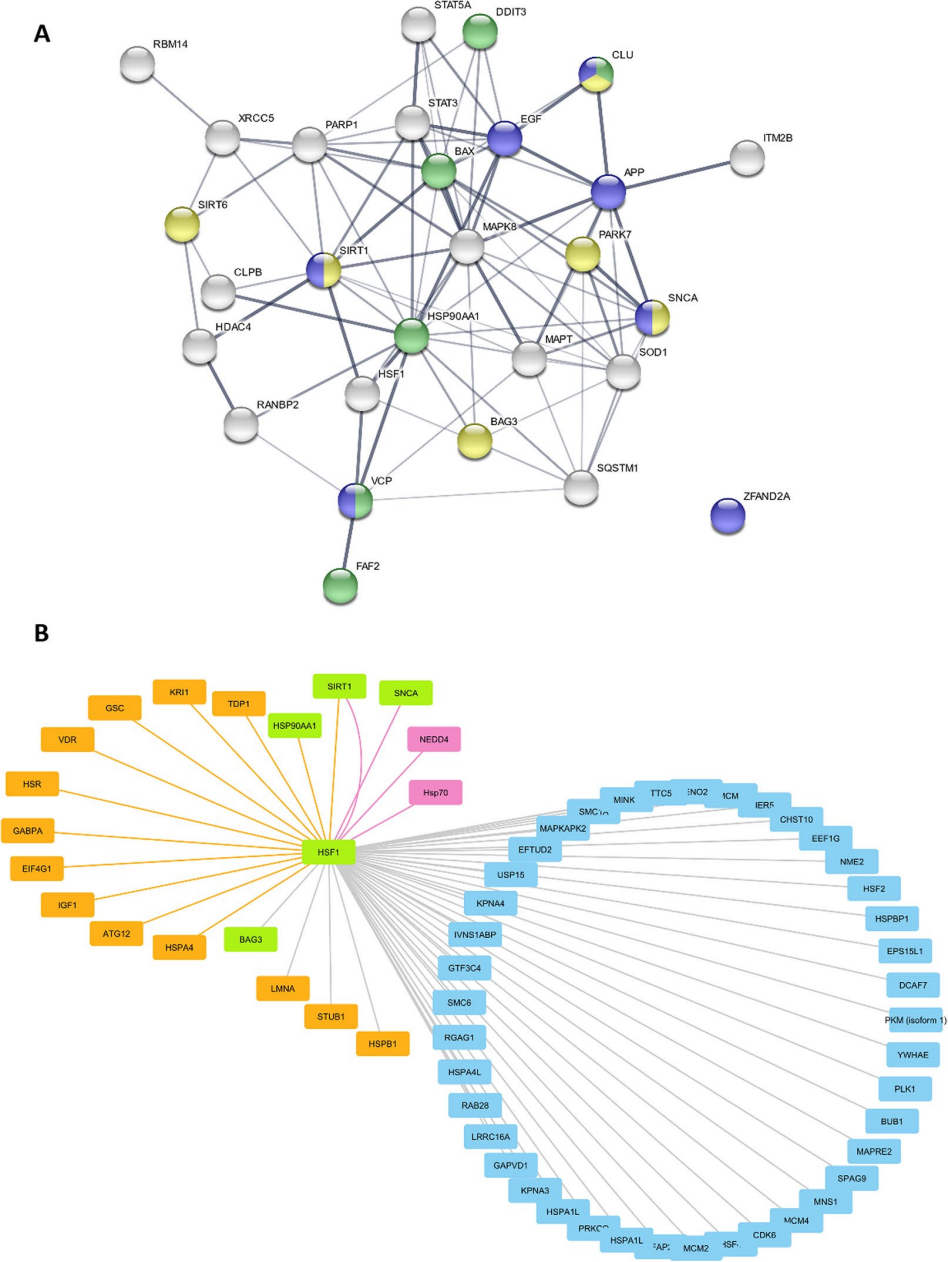

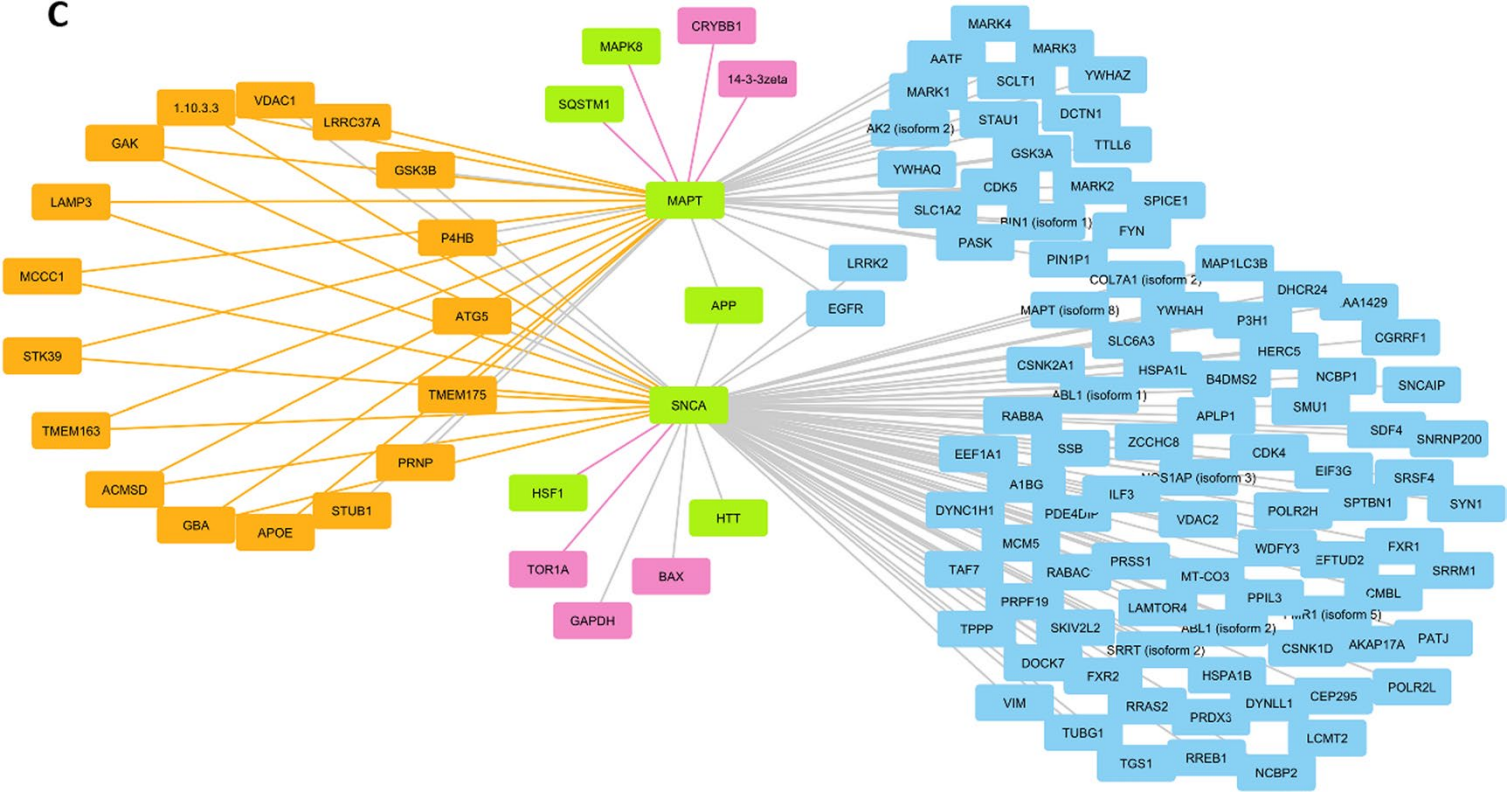
Subnetwork of proteins and protein–protein interactions identified by text-mining approaches as associated with proteostasis and aging. **A** Nodes (proteins) identified by both datasets were visualized using STRING and shown to participate in proteostasis-related processes, such as response to topologically incorrect protein (GO:0035966, green), regulation of protein stability (GO:0031647, yellow) or positive regulation of proteolysis (GO:0045862, blue). **B**, **C** Subnetworks centered in HSF1, and in MAPT and SNCA. Nodes (proteins) and Edges (interactions) from the automated and curated dataset networks are shown in orange and pink, respectively. Common proteins are represented in green. Nodes and edges retrieved from public databases are shown in blue and gray. The Group Attributes Layout based on the origin of the dataset (Curated, Automated or Databases) and the Prefuse Forced Directed Layout were used to visualize the network

After filtering from the text-mined PPIs the interactions already contained in the public databases, the curated network of PPIs contained 88 non-duplicated edges connecting 116 nodes. The same was done to the automated dataset of human proteins and a network of 323 nodes and 596 edges was obtained. Comparing these two, 28 of the identified proteins (nodes) were common to both datasets. Of note, 27 of these appear to directly interact and participate in a proteostasis-related network that has not been previously acknowledged (Fig. 1A). Functional enrichment of the 28 proteins present in both text-mining datasets indicates 13 significant clusters (Supplementary Table 1), mainly associated with protein degradation or folding, apoptosis and neuronal survival. Although with fewer genes, Alzheimer’s Disease and NAD-dependent ribosylation are among the clusters with higher fold enrichment (137.8% and 64.6%, respectively).

Considering only the proteins mapped to UniProt IDs, the two text-mining approaches together allowed the identification of 570 PPIs that were not present in the network obtained with database information. Examples are shown in Fig. 1B and 1C, representing the subnetworks centered in HSF1 and in α-synuclein (SNCA) and tau (MAPT). The subnetworks comprise all interactions obtained from the three different methods and show 61 HSF1 interactions, four identified by EGAS and 12 by Agilent (Fig. 1B). HSF1, NEDD4, SNCA, SIRT1 and their interactions can be retrieved from the subnetwork. In the second example (Fig. 1C), key proteins with a role in aging related disorders, such as Alzheimer’s disease and Parkinson’s disease are shown. Although most of the interactions are already found in the databases, EGAS and Agilent provided new information as well.

## Discussion

Proteostasis and aging seem to be closely related processes. Despite its most prevalent association to pathologies such as diabetes or neurodegenerative diseases, the aging process itself is associated with a severe decline in the proteostasis machinery. Here a text-mining strategy was followed, towards a most comprehensive overview of the molecular pathways involved both in proteostasis and in the aging process to identify predictive biomarkers. Two distinct but complimentary approaches were applied, using the Agilent Literature Search plugin for Cytoscape and EGAS that provide, respectively, automated and curated methods based on the analysis of the literature generated from terms of interest. Given the words that provide an association between proteostasis and aging as described in the methods section, networks of proteins were generated and further analyzed using Cytoscape, Reactome and PANTHER. The results demonstrate the valuable contribution of text-mining approaches to identify novel hypothesis and targets that can be further validated, as discussed next.

Considering the two text-mining strategies, the Agilent tool is less restrictive when compared to the manually curated approach and allows the use of full-text. On the other hand, manual literature curation results are more reliable, but since annotating full-text documents would require a much greater curation effort, only abstracts were annotated with EGAS. Accordingly, more information was retrieved with the automated method. Using EGAS, new information could still be extracted, namely, the identification of 82 PPIs that were not present in either the Agilent or the public databases networks.

Merging the networks from the two text-mining approaches revealed 28 common nodes (proteins) and 6 common edges (interactions). These included proteins such as PARK7, MAPK8, SQSTM1, SIRT1, VCP, STAT3, SIRT6, APP, DDIT3 and CLU, showing that these methods provide new, and literature supported information. For example, APP interaction with Superoxide Dismutase 1 (SOD1), a key participant in apoptotic signaling and oxidative stress, was detected. SOD1 had been associated with Amyotrophic lateral sclerosis (ALS) and was recently linked to Alzheimer’s disease (Muresan and Ladescu 2016). Clusterin also referred as apolipoprotein J (ApoJ), a small heat shock protein that acts as a molecular chaperone and promotes cell survival, has been largely reported in the literature associated with Alzheimer’s disease and other neurodegenerative diseases (Yuste-Checa et al. 2022; Jackson et al. 2019). According to the literature, CLU is a good target for the development of therapeutic approaches (Wilson and Zoubeidi 2017), however its interaction with APP was not found in the public databases network but was identified by the EGAS text-mining tool. In addition, both text-mining approaches identified the interaction of amyloid-β (APP) with BCL2 Associated Athanogene 3 (BAG3). BAG3 is involved in chaperone-assisted selective autophagy and was already associated to Alzheimer’s disease (Lei et al. 2015). Also, a BAG1 to BAG3 switch was observed during aging, being BAG1 associated with the removal of polyubiquitinated proteins from the proteasome and BAG3 related with the turnover of polyubiquitinated proteins from the autophagic-lysosomal system (Gamerdinger et al. 2009).

The enrichment analysis revealed the pathways that were overrepresented in our set of results. This means that there was a significant number of proteins/genes associated to those pathways, and this number is higher than we would have expected to find by chance, using the entire human genome as reference. The top 4 pathways were related to cellular responses to stress, particularly involving the HSF1 regulation or transactivation. EGAS provided two more HSF1 interactions with SNCA and NEDD4 that were not found in public databases. NEDD4 (Neural Precursor Cell Expressed, Developmentally Down-Regulated 4, E3 Ubiquitin Protein Ligase) is an enzyme that targets proteins for ubiquitination. Its involvement in neurodegeneration and interaction with HSF1 were described (Kim et al. 2016). Interestingly, the HSF1-SIRT1 interaction was acknowledged by both text-mining approaches. SIRT1, a NAD-dependent deacetylase well studied in aging, had a subnetwork with 136 edges, most of them found in databases (data not shown).

From the statistically significant processes represented, it should also be noted the overrepresentation of processes related to the immune system. The NLRP3 inflammasome is the major inflammatory complex (Broz and Dixit 2016) and its activation triggers the caspase-1 mediated processing of IL-1β and IL-18 into their bioactive cytokine forms and pyroptosis (Broz and Dixit 2016). It is not clear whether NLRP3 activation is a cause or a consequence of mitochondrial dysfunction however it is known that this complex can be activated through several damage-associated molecular patterns (DAMPs) such as ATP, DNA, heat shock proteins and protein aggregates or even mitochondrial DAMPs such as cathepsin B and reactive oxygen species (ROS) (Gurung et al. 2015). Moreover, the activation of mitochondrial biogenesis that is a pathway related to the inflammasome activation is also observed. NLRP3 activation is enhanced in many age-related diseases and is also associated with aging itself (Gritsenko et al. 2020). The acetylation status of NLRP3 is affected during aging. NLRP3 is normally modified by SIRT2-dependent deacetylation in macrophages, a process that is dysregulated during aging. This switch towards an acetylated status activates the inflammasome leading to chronic inflammation and insulin resistance (He et al. 2020).

## Conclusion

In conclusion, the work presented here shows how text-mining tools can be used to extract knowledge from available data. To face the increasing number of published papers in a topic such as aging and the need to progress with directed studies to identify new potential biomarkers or therapeutic targets, text-mining tools provide a useful approach as a first step in the research process.

## Supplementary Information

Below is the link to the electronic supplementary material.

Supplementary file1 (PPTX 2531 kb)

## Data Availability

Not applicable.
