# Supplementary material for: Proteostasis networks in aging: novel insights from text-mining approaches
Source: Biogerontology. 2023 Apr 1;24(4):555–62. doi: 10.1007/s10522-023-10027-0 (PMC10267007; doi:10.1007/s10522-023-10027-0)
Supplement: Supplementary file 1 — Supplementary file1 (PPTX 2531 kb) [file 10522_2023_10027_MOESM1_ESM.pptx]

## Slide 1
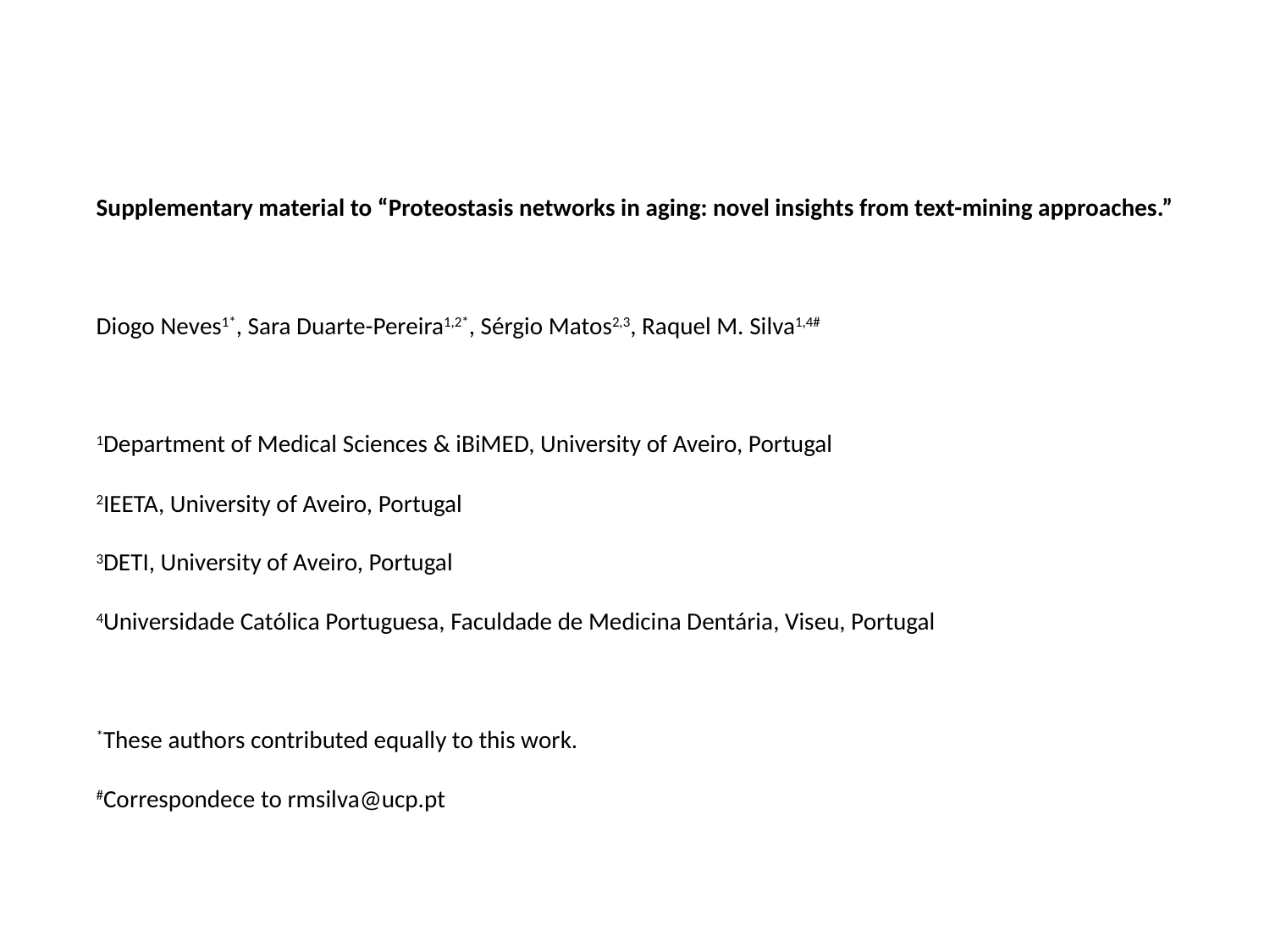

Supplementary material to “Proteostasis networks in aging: novel insights from text-mining approaches.”
Diogo Neves1*, Sara Duarte-Pereira1,2*, Sérgio Matos2,3, Raquel M. Silva1,4#
1Department of Medical Sciences & iBiMED, University of Aveiro, Portugal
2IEETA, University of Aveiro, Portugal
3DETI, University of Aveiro, Portugal
4Universidade Católica Portuguesa, Faculdade de Medicina Dentária, Viseu, Portugal
*These authors contributed equally to this work.
#Correspondece to rmsilva@ucp.pt

## Slide 2
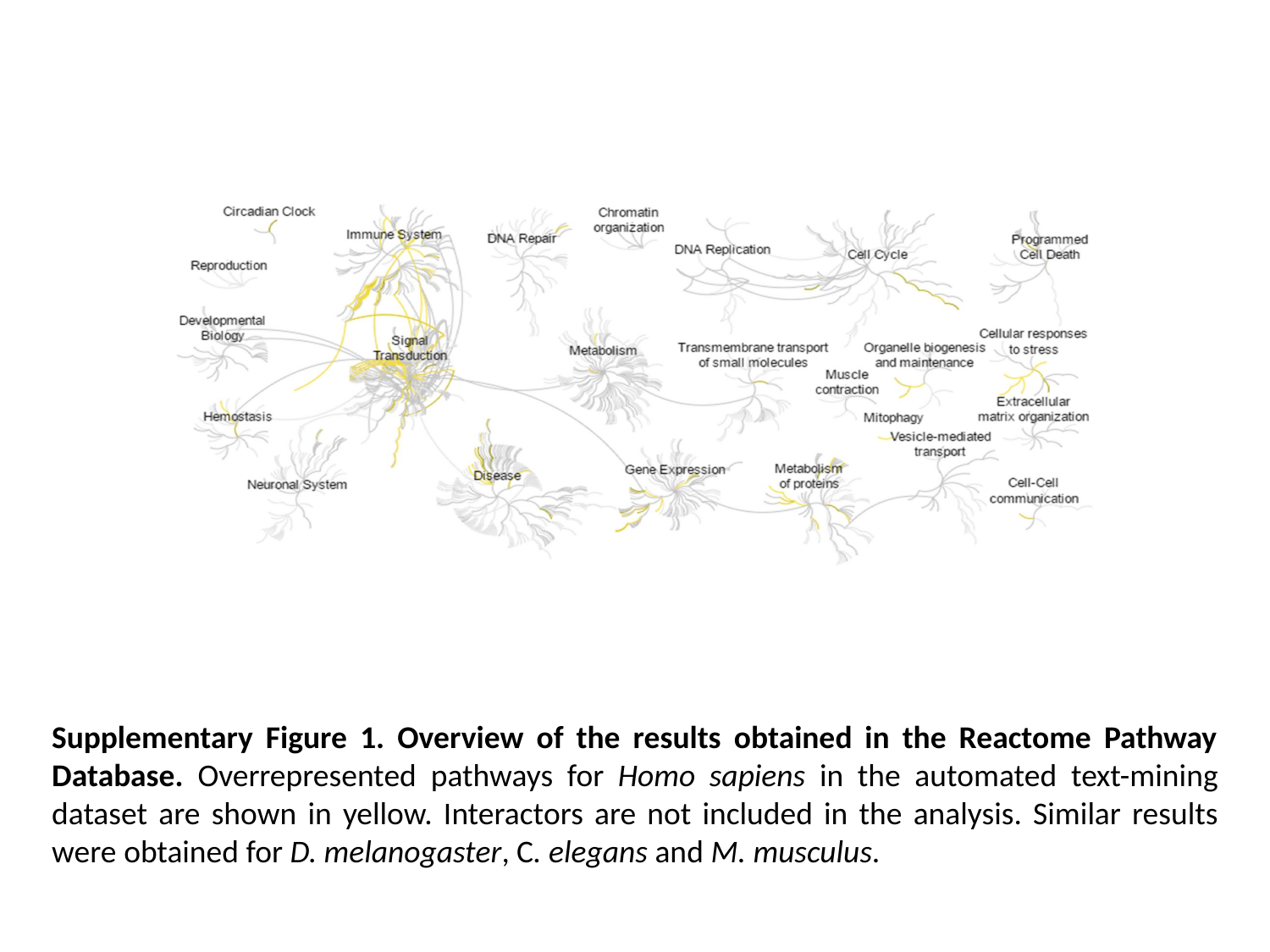

Supplementary Figure 1. Overview of the results obtained in the Reactome Pathway Database. Overrepresented pathways for Homo sapiens in the automated text-mining dataset are shown in yellow. Interactors are not included in the analysis. Similar results were obtained for D. melanogaster, C. elegans and M. musculus.

## Slide 3
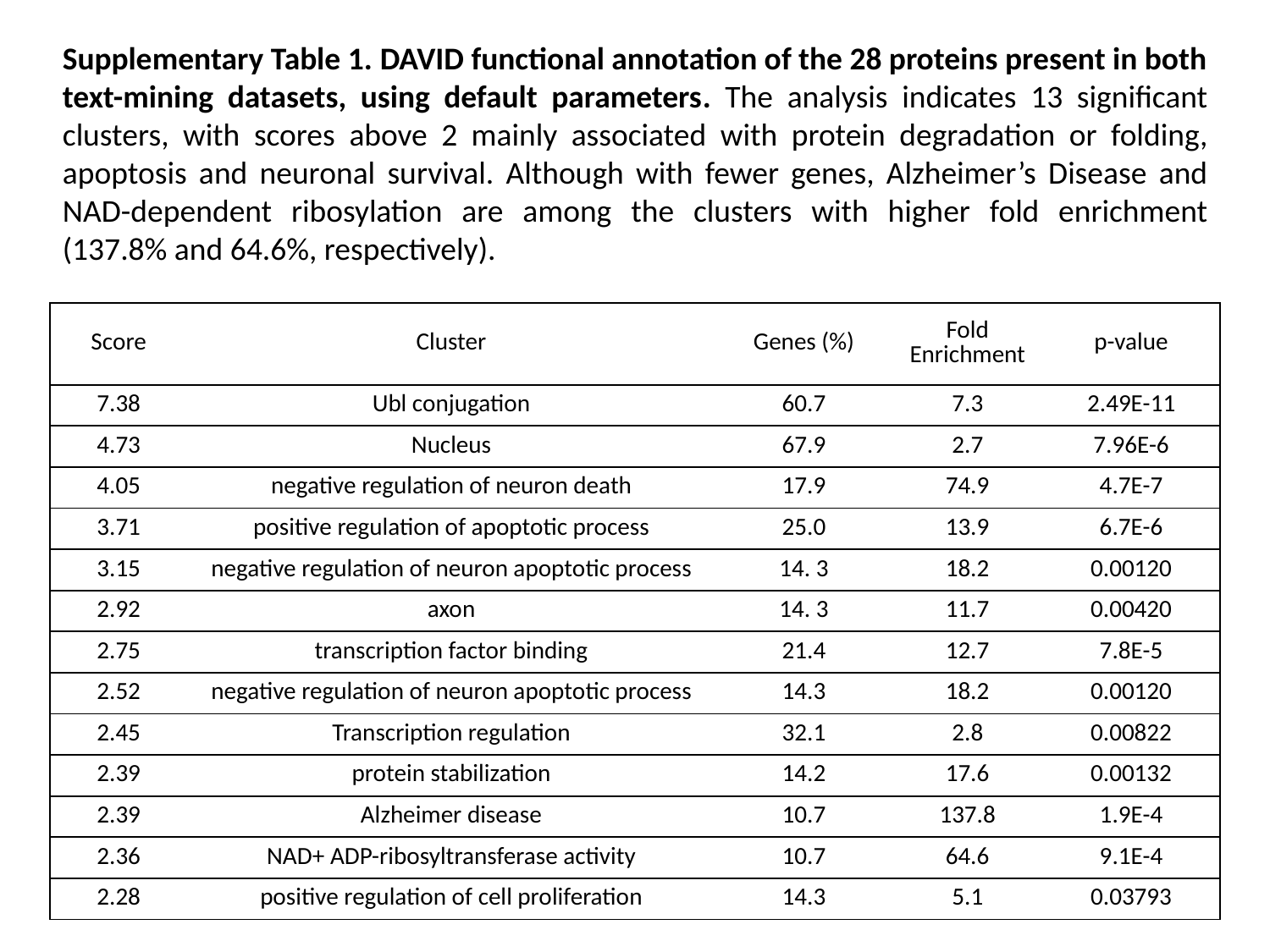

Supplementary Table 1. DAVID functional annotation of the 28 proteins present in both text-mining datasets, using default parameters. The analysis indicates 13 significant clusters, with scores above 2 mainly associated with protein degradation or folding, apoptosis and neuronal survival. Although with fewer genes, Alzheimer’s Disease and NAD-dependent ribosylation are among the clusters with higher fold enrichment (137.8% and 64.6%, respectively).
| Score | Cluster | Genes (%) | Fold Enrichment | p-value |
| --- | --- | --- | --- | --- |
| 7.38 | Ubl conjugation | 60.7 | 7.3 | 2.49E-11 |
| 4.73 | Nucleus | 67.9 | 2.7 | 7.96E-6 |
| 4.05 | negative regulation of neuron death | 17.9 | 74.9 | 4.7E-7 |
| 3.71 | positive regulation of apoptotic process | 25.0 | 13.9 | 6.7E-6 |
| 3.15 | negative regulation of neuron apoptotic process | 14. 3 | 18.2 | 0.00120 |
| 2.92 | axon | 14. 3 | 11.7 | 0.00420 |
| 2.75 | transcription factor binding | 21.4 | 12.7 | 7.8E-5 |
| 2.52 | negative regulation of neuron apoptotic process | 14.3 | 18.2 | 0.00120 |
| 2.45 | Transcription regulation | 32.1 | 2.8 | 0.00822 |
| 2.39 | protein stabilization | 14.2 | 17.6 | 0.00132 |
| 2.39 | Alzheimer disease | 10.7 | 137.8 | 1.9E-4 |
| 2.36 | NAD+ ADP-ribosyltransferase activity | 10.7 | 64.6 | 9.1E-4 |
| 2.28 | positive regulation of cell proliferation | 14.3 | 5.1 | 0.03793 |
